# Elevated expression of RUNX3 co-expressing with EZH2 in esophageal cancer patients from India

**DOI:** 10.1186/s12935-020-01534-y

**Published:** 2020-09-10

**Authors:** Asad Ur Rehman, Mohammad Askandar Iqbal, Real Sumayya Abdul Sattar, Snigdha Saikia, Mohammad Kashif, Wasif Mohammad Ali, Subhash Medhi, Sundeep Singh Saluja, Syed Akhtar Husain

**Affiliations:** 1 Department of Biosciences, Jamia Millia Islamia, New Delhi, 110025 India; 2 Department of Biotechnology, Jamia Millia Islamia, New Delhi, India; 3 Department of Bioengineering and Technology, Guist, Gauhati University, Guwahati, India; 4 National Institute of Immunology, New Delhi, India; 5 Department of Surgery, JN Medical College and Hospital, AMU, Aligarh, UP India; 6 Department of Gastrointestinal Surgery, G B Pant Hospital & Maulana Azad Medical College, New Delhi, India

**Keywords:** RUNX3, EZH2, Esophageal cancer, DNA methylation

## Abstract

**Background:**

Runt related transcription factor3 (RUNX3) is considered as a tumor suppressor gene (TSG) that functions through the TGF-β dependent apoptosis. Promoter methylation of the CpG islands of RUNX3 and overexpression of enhancer of zeste homolog 2 (EZH2) has been suggested to downregulate RUNX3 in cancer.

**Methods:**

Here, we studied the expression of RUNX3 and EZH2 in 58 esophageal tumors along with paired adjacent normal tissue. mRNA levels, protein expressions and cellular localizations of EZH2 and RUNX3 were analyzed using real-time PCR and immunohistochemistry, respectively. DNA methylation was further assessed by the methylation specific-PCR.

**Results:**

Compared to normal tissue, a significant increase in expression of RUNX3 mRNA in 31/57 patient’s tumor tissue (*p* < 0.04) was observed. The expression of EZH2 was found to be upregulated compared to normal, and a significant positive correlation between EZH2 and RUNX3 expression was observed (*p* = 0.002). 22 of the 27 unmethylated cases at the promoter region of the RUNX3 had elevated RUNX3 protein expression (*p* < 0.001).

**Conclusion:**

The data presented in this study provide new insights into the biology of RUNX3 and highlights the need to revisit our current understanding of the role of RUNX3 in cancer.

## Background

With increasing environmental stresses and unhealthy lifestyles, cancer has become a bane for humans with around 14.1 million new cancer cases turning up. Thousands of people every year dwell with one of the hundred types of cancer and it has been estimated that around 8.2 million people die due to cancer [1]. Esophageal cancer (CaEs), a cancer of the gastrointestinal tract has, become eighth most common cancer worldwide and, leads at sixth position in context of the deaths due to cancers [2]. 450,000 people worldwide are currently suffering from CaEs which exists majorly as esophageal squamous cell carcinoma (ESCC) and adenocarcinoma (EAC) [3, 4]. Adenocarcinoma occurs mainly in Western countries and often preceded with the GERD whereas ESCC found to be the predominant type of CaEs in Asia pacific region [5]. Treatment includes surgery, chemotherapy and radiotherapy which are either given separately or in combination with one another. However, surgery is the most opted therapy for esophageal tumor [6]. The overall prognosis of CaEs is poor with 5-year survival rates ranging between 15 and 50% [7, 8]. Therefore, there is an urgent need to identify putative targets of clinical relevance.

It is well-known that genetic mutations in the tumor suppressor and/or proto-oncogenes are responsible for initiation and progression of cancer [9–11]. In CaEs, many tumor suppressor genes (TSGs) have been discovered [12–14]. Recently, the runt-related transcription factor 3 gene (RUNX3), belonging to the runt domain family of transcription factors, has gained attention for its role in tumor progression [15]. A broader consideration revealed its conjunction with the TGF-β pathway and its upregulation which induce cell cycle arrest, apoptosis and bring down cyclin D1 expression [16–19]. Studies have indicated a tumor-suppressing role of RUNX3 [20] and complete inactivation or downregulation of RUNX3 gene has been associated with gastric cancer [17], CaEs [21, 22] pancreatic cancer [23]. RUNX3 inactivation or downregulation has shown to be dictated by hemizygous deletion [17] or mislocalization [24] or hypermethylation [25]. However, some evidence suggests that RUNX3 may have oncogenic role in cancer [26]. Accordingly, the current study attempts to investigate the hitherto unknown status of RUNX3 in Indian esophageal cancer patients.

Enhancer of zeste homolog 2 (EZH2) is known to down-regulate the expression of RUNX3. EZH2, a histone methyl transferase, is a member of polycomb group of genes (Pcg) [27]. Frequent EZH2 over-expression has been associated with cancer, however, the underlying mechanism remains unelucidated [28–30]. Furthermore, EZH2 is known to down-regulate the expression of RUNX3 in gastric cancers [31]. In addition, hypermethylation of RUNX3 promoter has been associated with down-regulation of RUNX3 gene expression in cancers [31, 32]. We therefore, studied the status of EZH2 and its correlation with RUNX3 expression in Indian esophageal cancer patients.

## Material and methods

### Selection of patient material

Tumor samples were collected from 58 esophageal cancer patients were procured from the Department of Gastrointestinal Surgery, G.B. Pant Hospital between December 2013 and March 2017. The cases selected were based on the following criteria: (i) histological proven primary ESCC with available biopsy specimens; (ii) no previous malignant disease or a second primary tumor; (iii) no previous treatment or severe complications; (iv) no chemotherapy or radiotherapy given; (v) patient belonged to the North Indian region. All the other patients who does not follow the above criterion were excluded from the study.

Recruited patient’s tumor tissue specimens were taken by surgical resection as well as from endoscopic biopsy depending on the treatment which was given to the concerned patient. Adjacent normal esophageal mucosa from same patient was used as control. The clinicopathological factors were taken into the account and every patient was followed till May 2017. Written consent letters were obtained before the tissue excision was carried out. The study was approved by the medical ethics committee of Jamia Millia Islamia as well as G.B. Pant Hospital.

### Real-time PCR (qPCR)

Total RNA was isolated from ESCC tissues and the corresponding normal tissues stored in RNA later (Qiagen) using Trizol reagent (Invitrogen, Carlsbad, California, USA), and reverse transcribed into cDNA (1.2 μg) with iscript™ Reverse Transcription Reagents (Bio-Rad laboratories Inc.). PCR was performed with lightcycler^®^ 96 SYBR Green I Master (Roche Diagnostics India Pvt Ltd) by using primers for RUNX3 (15): sense 5-GACTGTGATGGCAGG CAATGA-3 and antisense 5-CGAAGCGAAGGTCGTTGAA-3, which amplify a 101 bp product and for EZH2: sense 5-ACGTCAGATGGTGCCAGCAATA-3 and antisense 5-CCCTGACCTCTGTCTTACTTG TGGA-3, which amplify a 120 bp product. The *β*-Actin mRNA was also amplified as an internal control using the following primers: sense 5-AGATGTGGATCAGCAAGC AG-3 and antisense 5-GCGCAAGTTAGGTTTTGTCA-3, which amplify a 122 bp product. The real time PCR was performed on the similar lines as carried out previously [33]. Amplification cycles consisted of denaturation at 95 °C for 1 min, 35 cycles of denaturation at 94 °C for 20 s, annealing at 59 °C for 15 s, extension at 72 °C for 20 s, and a final elongation at 72 °C for 7 min. Measurements were performed in triplicates. The relative amount of mRNA was calculated as the calibrator normalized ratio using lightcycler 96 Software 1.5. The calibrator normalized ratio was measured as the following formula:

$${\text{RQ}} = 2^{- {\Delta \Delta } {\text{Ct}}},  {\Delta \Delta }{\text{Ct}}  = ({\text{Ct}}_{\text{targeted gene}}  - {\text{Ct}}_{{ \upbeta}{\text{-actin}}}) \; { \text{targeted sample}} - ({\text{Ct}}_{\text{targeted gene}}  - {\text{Ct}}_{{ \upbeta}{\text{-actin}}}) \; { \text{calibration sample}}.$$

### Genomic DNA extraction

High molecular weight genomic DNA was extracted from above specimens by using genomic DNA extraction kit (MDI India) as per the manufacturer's instructions. The quantity and quality of the DNA was analyzed by Nanodrop ND1000 spectrophotometer and later by running on the 1% agarose gel stained with ethidium bromide.

### Methylation specific PCR (MS-PCR)

Methylation specific PCR was done as reported earlier [34]. All samples gDNA then were subjected to bisulfite conversion using the EZ DNA Methylation kit or the EZ DNA Methylation-Lightning kit (Zymo Research), by following the instruction given by the manufacturer. Bisulfite converted DNA was amplified by two different sets of primers specific to unmethylated and methylated RUNX3 sequences. The primers were designed using Methprimer tool [35]. The primer pairs for the methylated detection were in the RUNX3 promoter region: sense 5GGTTTAGTTAATGAGTTAAGGTCGC-3 and antisense 5-TCTAATAAATACGAAAACG ACCGA-3, which amplify a 193 bp product; for the unmethylated detection the primers were: sense 5-TTTAGTTAATG AGTTAAGGTTGTGA-3 and antisense 5-TCTAATAAATACAAAAACAACCAAA-3, which amplify a 190 bp product. For positive control, commercially available completely methylated and unmethylated human genomic DNA were taken whereas, double distilled water was used in place of bisulfite converted DNA for negative control. The PCR was performed in 25 µl reaction volume containing 100 ng of bisulfite converted DNA, 1.5 mM MgCl_2,_ 200 µM of each dNTPs, 0.5 µM each of forward and reverse oligonucleotides primers, 1× PCR buffer and 1 unit of Hot Start Taq polymerase (Qiagen, Valencia, CA) hot start master mix and consisted of 35 cycles at 96 °C for 20 s; 56 °C/53 °C for 20 s; and 72 °C for 30 s after the initial denaturation step (94 °C for 5 min). A final extension was at 72 °C for 10 min. Aliquots from PCR products were visualized on 2% agarose gel containing ethidium bromide, analyzed and photographed using Gel Doc (Bio-Rad Laboratories, CA, USA) under UV illumination. As an internal quality control, each MSP was repeated and no discordant results were obtained.

### Immunohistochemistry (IHC)

IHC was performed as reported earlier [36]. IHC Staining was carried on formaline fixed tissue samples. The tissue was embedded in paraffin and then cut into 4–5 µm tissue sections which were then taken on Poly-l-lysine coated slides. Xylene with differential grades of ethanol led to the deparaffinization of the tissue samples. Internal peroxidase activity was quenched by the application of 0.3% H_2_O_2_ for 30 min and subsequent 100 °C citrate buffer at pH 9 was done for Ag retrieval. Sections were blocked with TENG-T [10 mM Tris, 5 mM Ethylenediaminetetraacetic acid, 0.15 mol/l NaCl, 0.25% gelatin, 0.05% (vol/vol) Tween 20, pH 8.0] for 30 min. Slides were incubated with primary antibodies to EZH2 (1:5000) and to RUNX3 (1:4000) overnight at 4 °C in phosphate-buffered saline with 0.1% Triton and 1% bovine serum albumin. Afterwards, incubation with secondary biotinylated secondary antibody against mouse and rabbit and streptavidin horse-radish peroxidase were carried out each for 20 min. DAB was added to visualize the antibody antigen reaction, and counterstained with hematoxylin. Normal esophagus tissue was used s positive control and negative control sections for all antibodies were processed in an identical manner after omitting the primary antibody and showed no staining. staining was then interpreted by expert pathologists under light microscope at 400X magnification.

The degree of immunoreactivity of both EZH2 and RUNX3 was categorized as follows: High reactivity, more than 50% of cells showing intense immunoreactivity in their nuclei; Low reactivity, 50% of fewer cells showing intense immunoreactivity in their nuclei. The mean percentage of positive tumor cells was determined in at least five areas at high power field [37].

### Statistical analysis

Data are expressed as mean ± standard deviation (SD). Statistical analysis was performed using the Statistical Package of Social Science (SPSS). The chi-square test and Fisher’s exact test were used where appropriate. The Wilcoxon signed-ranks test and Kruskal–Wallis test were drawn to assess the significance in differences at the expression levels of RUNX3/β-Actin mRNA. Spearman’s rank correlation coefficient was calculated to analyze the association between EZH2 and RUNX3 messenger RNA (mRNA) expression. *p*-values < 0.05 were considered as significant.

## Results

### Upregulated RUNX3 mRNA expression in esophageal tumors

**Fig. 1:**
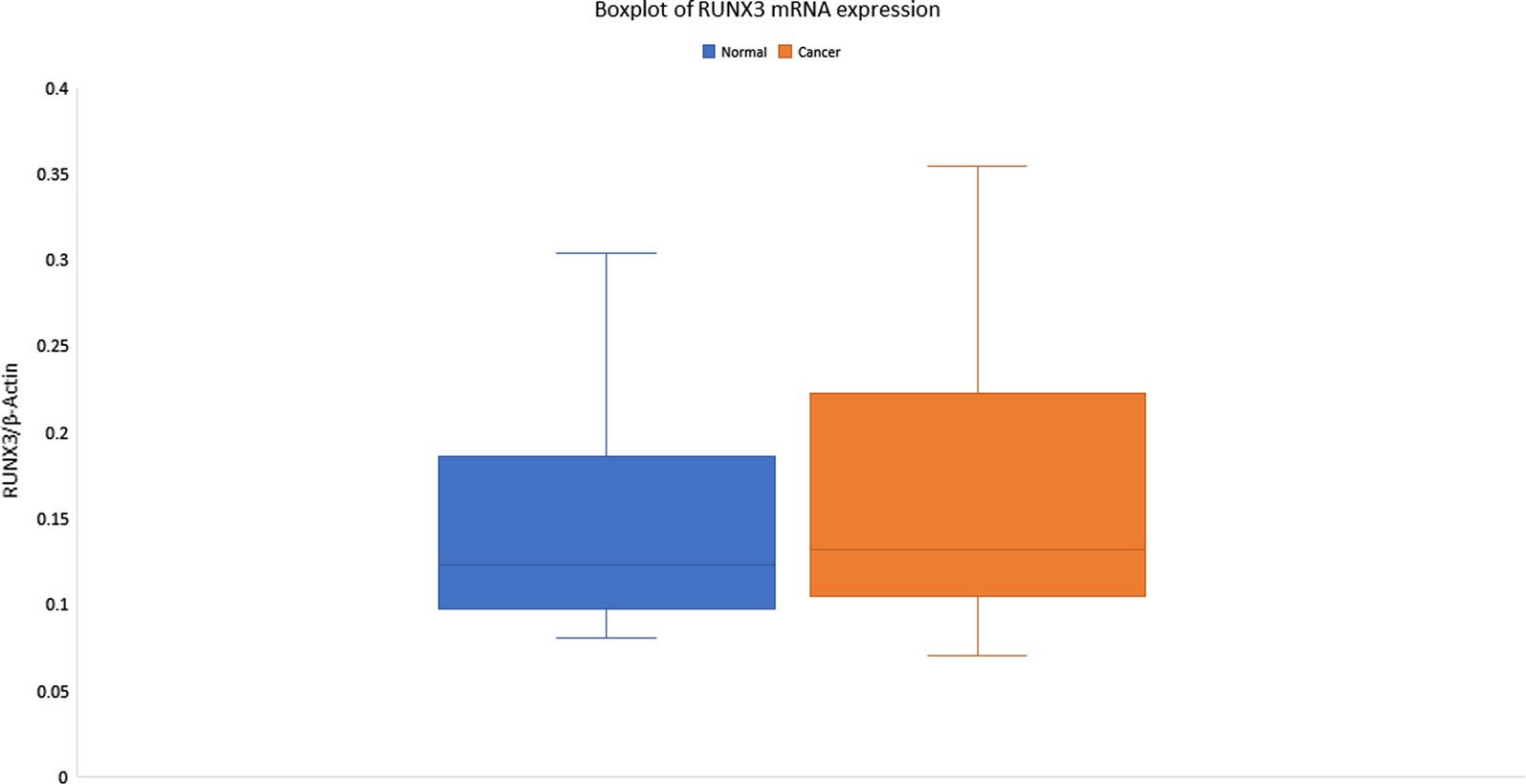
Real-time PCR analysis of RUNX3 in esophageal cancer patients

Real-time PCR was performed on cDNA from 57 CaEs tumor and adjacent normal tissues, expression of RUNX3 mRNA was found to be significantly increased in tumors (5.056 ± 5.331, relative values to β-actin expression) compared with normal tissue (5.603 ± 5.709 relative values to β-actin expression) (*p* < 0.04) (Fig. 1). The overall mean fold change was found to be up-regulated by 5.15 ± 10.05-fold. However, when RUNX3 upregulation was correlated with the different grades of dysphagia, no significant association was seen (*p* < 0.38), and the degree of association was found to be very weak. Also, no significant correlation of RUNX3 with different clinicopathological parameters was found (Table 1).

**Table 1:** Correlation of RUNX3 mRNA expression with the clinicopathological factors of esophageal cancer patients

Clinicopathological parameters	No. of patients	RUNX3 expression relative to β-Actin	*p-*value
Age			
≥ 50	44	4.26 ± 4.9	0.351
< 50	13	5.28 ± 5.4	
Sex			
Male	32	5.34 ± 4.4	0.711
Female	25	4.68 ± 6.4	
TNM Classification			
Locally advanced resectable	28	5.05 ± 5.8	
Locally advanced unresectable	25	5.04 ± 5.2	0.728
Metastatic	4	5.13 ± 2.8	
Dysphagia Grade			
dys Gr1	1	4.09 ± 0	
dys Gr2	19	5.25 ± 5.8	
dys Gr3	24	4.92 ± 5.2	0.359
dys Gr4	11	6.48 ± 3.1	
dys Gr5	2	-2.6 ± 9.05	
Location of Tumor			
Upper third	7	7.88 ± 3.1	
Middle third	25	4.78 ± 4.6	0.272
Lower third	25	4.53 ± 6.2	
Smoking			
Smoker	32	5.46 ± 4.7	0.742
Non-smoker	25	4.53 ± 6.04	
Alcohol			
Alcoholic	21	5.5 ± 3.7	0.967
Non-Alcoholic	36	4.79 ± 6.1	
Tobacco			
Tobacco Chewer	5	7.62 ± 4.1	0.438
Non-Tobacco Chewer	52	4.81 ± 5.4	
Type			
SCC	51	5.12 ± 5.5	0.153
Adeno	6	4.45 ± 2.6	
Diet			
Non Veg	31	5.39 ± 4.1	0.866
Veg	26	4.77 ± 6.2	

### Absence of promoter DNA methylation correlated with upregulated RUNX3

**Fig. 2:**
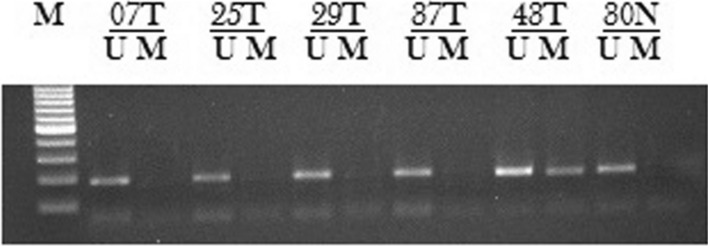
Methylation status of RUNX3 in esophageal cancer patients. DNA methylation was assessed by using two specifically designed primers to amplify either methylated DNA (*M*) or unmethylated DNA (*U*). N: Normal tissue; T: Tumor tissue

In our study, we found unmethylated CpG in RUNX3 in 47.36% (27/57) samples and out of these 27 samples 81.48% (22/27) samples showed elevated expression of the RUNX3 protein in the tumor as compared to the normal tissue (Fig. 2). Whereas, MSP analysis also pointed to DNA aberrant methylation in 52.63% (30/57) of the CaEs patients and out of these only 9 samples showed upregulation. Hence, a significant correlation was seen between CpG methylation and the RUNX3 expression (*p* < 0.001). Also, we found that in 06 cases with methylation as well as up-regulated EZH2 protein expression RUNX3 was downregulated in tumor tissue. Whereas, 04 cases had reduced RUNX3 protein expression without corresponding methylation and 02 cases among them had up-regulated EZH2 (Table 2).

**Table 2:** Correlation between RUNX3 methylation and mRNA expression

In North Indian population	No. of methylated samples	No. of unmethylated samples	*p-*value
Downregulated RUNX3	21	05	< 0.001
Upregulated RUNX3	9	22	

### RUNX3 mRNA expression positively correlated with EZH2 mRNA and protein level

**Fig. 3:**
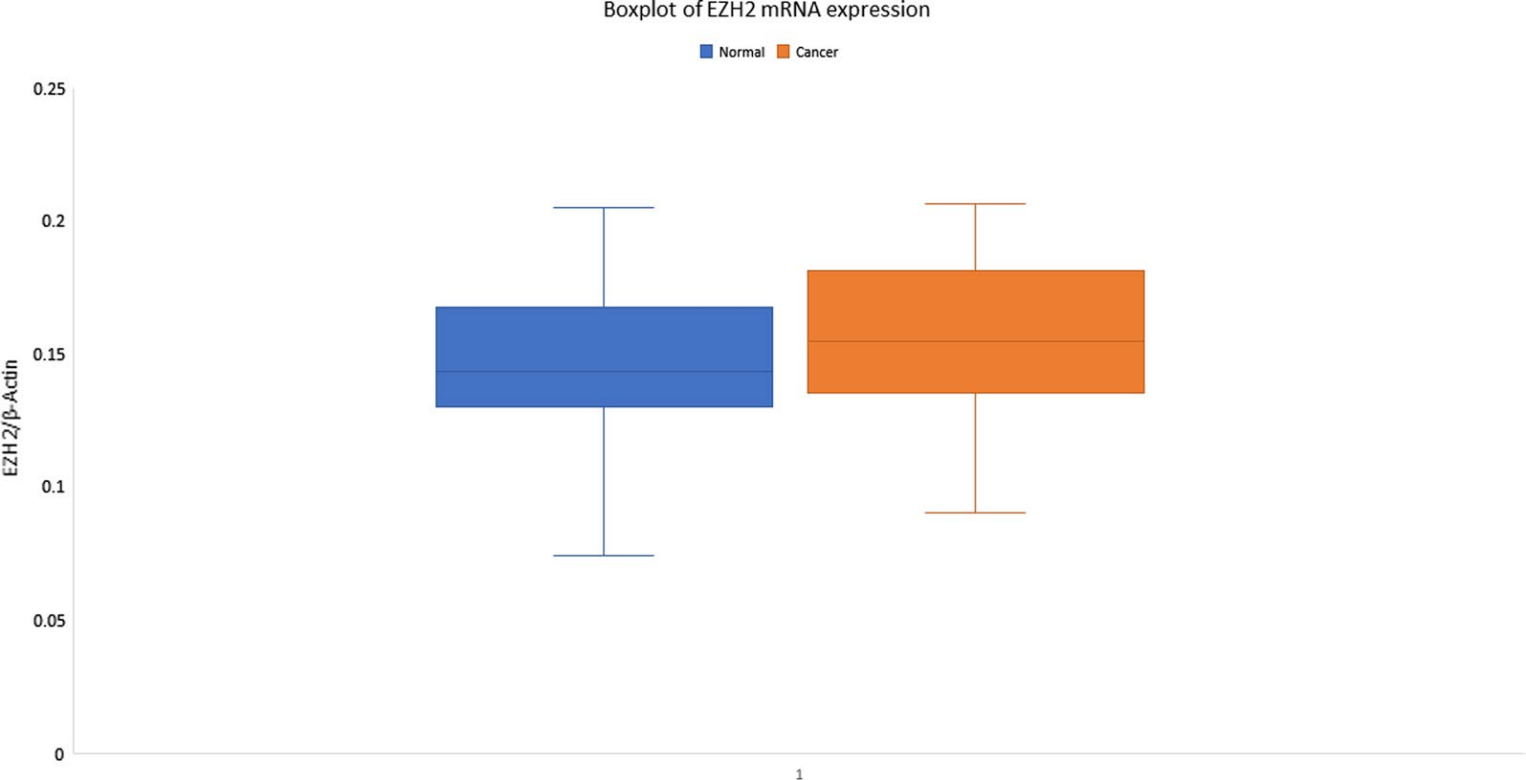
Real-time PCR analysis of EZH2 in esophageal cancer patients

The mean fold change of expression of EZH2 mRNA was found to be > twofold up-regulated in 52 samples where expression of EZH2 was seen. The expression of EZH2 was increased in tumors (6.551 ± 1.527, relative values to B-actin expression) compared with normal tissue (6.565 ± 2.139 relative values to B-actin expression) (Fig. 3). Also, no significant association was observed between the EZH2 expression and the dysphagia grade, the degree of association also was found to be very weak (Table 3). A positive correlation was observed between the mRNA expression status of EZH2 and RUNX3. Out of 22 cases with downregulated RUNX3 protein expression 08 cases had upregulated EZH2 protein expression and 14 cases had shown downregulation (Table 4). Whereas in 23 cases of RUNX3 upregulation had EZH2 up regulation and in 06 cases downregulation of EZH2 was observed (*p* = 0.002).

**Table 3:** Correlation of EZH2 mRNA expression with the clinicopathological factors of esophageal carcinoma patients

Clinicopathological parameters	No. of patients	EZH2 expression relative to β-Actin	*p-*value
Age			
≥ 50	40	6.53 ± 1.4	0.595
< 50	12	6.61 ± 1.9	
Sex			
Male	31	6.47 ± 1.4	0.751
Female	21	6.66 ± 1.6	
Classification			
Locally advanced resectable	25	6.59 ± 1.5	
Locally advanced unresectable	23	6.63 ± 1.6	0.532
Metastatic	4	5.79 ± 1.1	
Dysphagia Grade			
dys Gr1	1	6.34 ± 0	
dys Gr2	18	6.31 ± 1.5	
dys Gr3	21	6.96 ± 1.5	0.314
dys Gr4	11	6.41 ± 1.2	
dys Gr5	1	3.83 ± 0	
Location of Tumor			
Upper third	7	6.28 ± 1.9	
Middle third	24	6.67 ± 1.6	0.799
Lower third	21	6.51 ± 1.2	
Smoking			
Smoker	30	6.51 ± 1.3	0.803
Non-smoker	22	6.60 ± 1.7	
Alcohol			
Alcoholic	20	6.58 ± 1.6	0.94
Non-Alcoholic	32	6.53 ± 1.5	
Tobacco			
Tobacco Chewer	5	6.32 ± 1.2	0.78
Non-Tobacco Chewer	47	6.57 ± 1.5	
Type			
SCC	47	6.58 ± 1.5	0.609
Adeno	5	6.19 ± 0.8	
Diet			
Veg	28	6.33 ± 1.3	0.233
NonVeg	24	6.80 ± 1.6

**Table 4:** Correlation between RUNX3 mRNA expression and EZH2 mRNA expression

In North Indian population	Downregulated EZH2	Upregulated EZH2	*p-*value
Downregulated RUNX3	14	8	0.002
Upregulated RUNX3	6	23	

^*^Pearson Chi-square test

### Subcellular localization of RUNX3 and EZH2 was found to be predominantly in nucleus

**Fig. 4:**
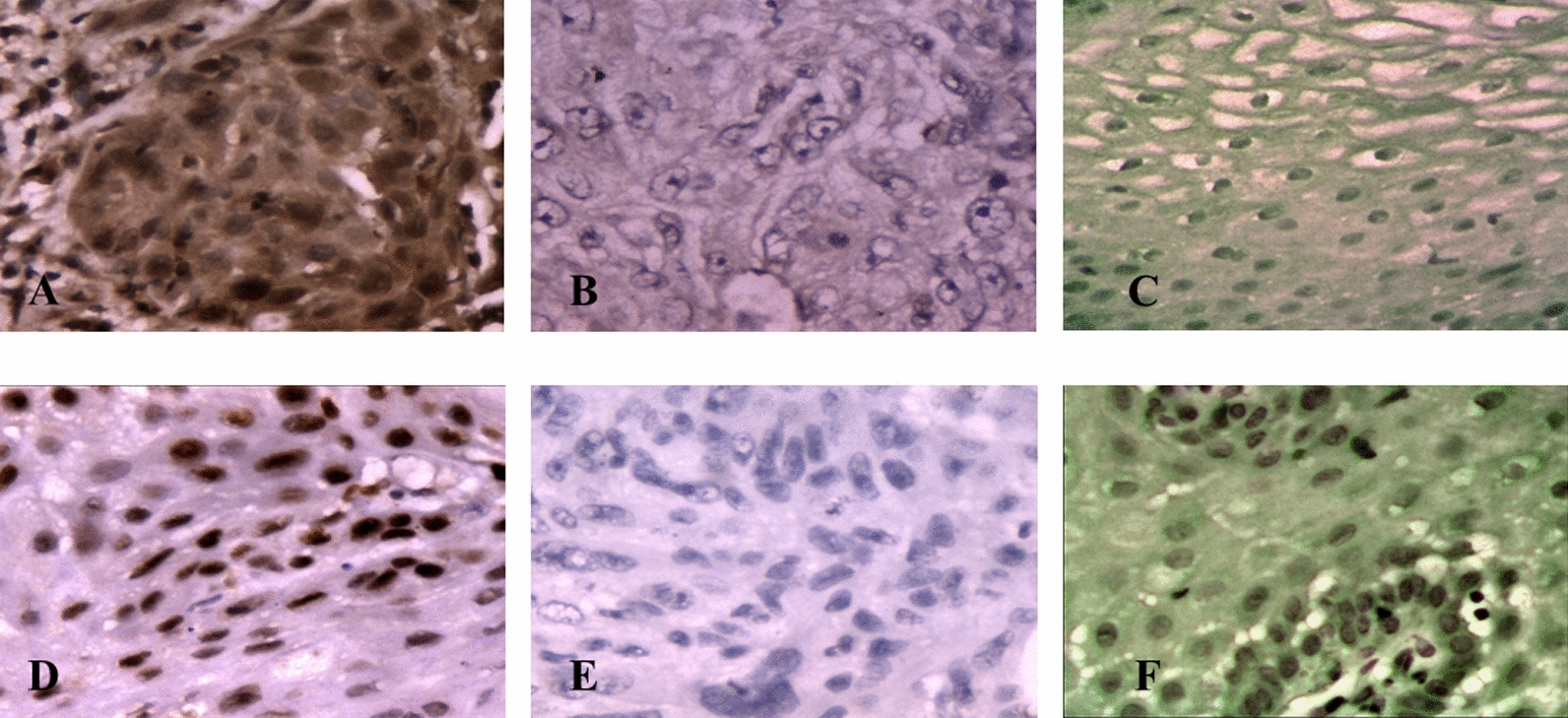
Expression of RUNX3 and EZH2 as detected by IHC: **a** EZH2 high expression in esophageal tumor tissue. **b** EZH2 low expression in esophageal tumor tissue. **c** EZH2 expression in normal esophageal tissue. **d** RUNX3 high expression in tumor esophageal tissue. **e** Low RUNX3 expression in esophageal tumor tissue. **f** RUNX3 expression in normal esophageal tissue

57 samples were tested for the RUNX3 and 26 samples showed low reactivity whereas 31 samples showed moderate to high reactivity. EZH2 was assessed for its expression and 21 cases were found to have low expression and in 31 cases moderate to high expression was observed (Fig. 4). These results again very well corroborated with the real time mRNA expression. All the positive cases showed nuclear expression for RUNX3 and EZH2.

### Elevated expression of RUNX3 as revealed by oncomine database

**Fig. 5:**
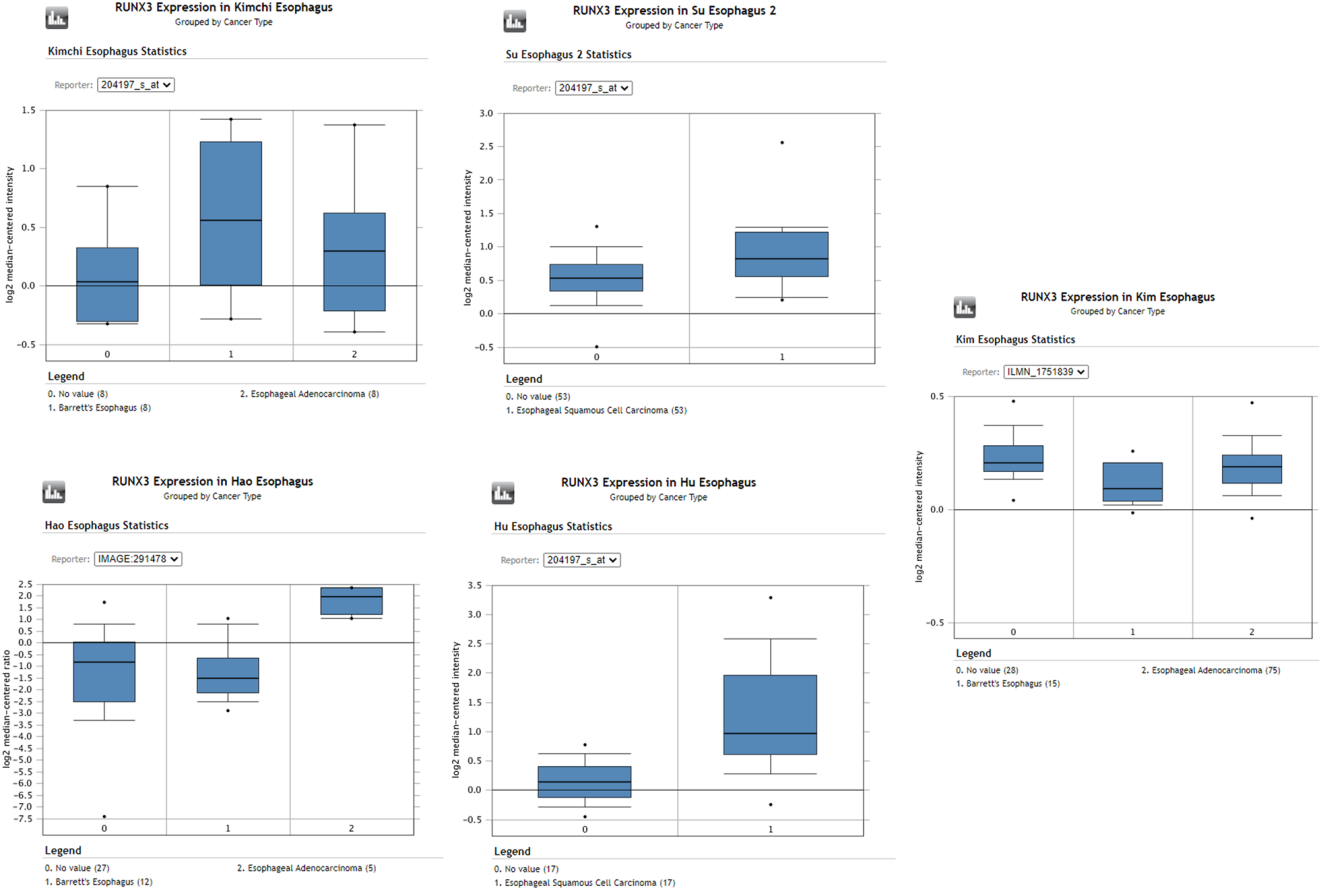
RUNX3 expression from oncomine dataset. Box plots from Oncomine representing the higher RUNX3 expression in Esophageal adenocarcinoma and Esophageal squamous cell carcinoma, compared to normal esophagus

**Fig. 6:**
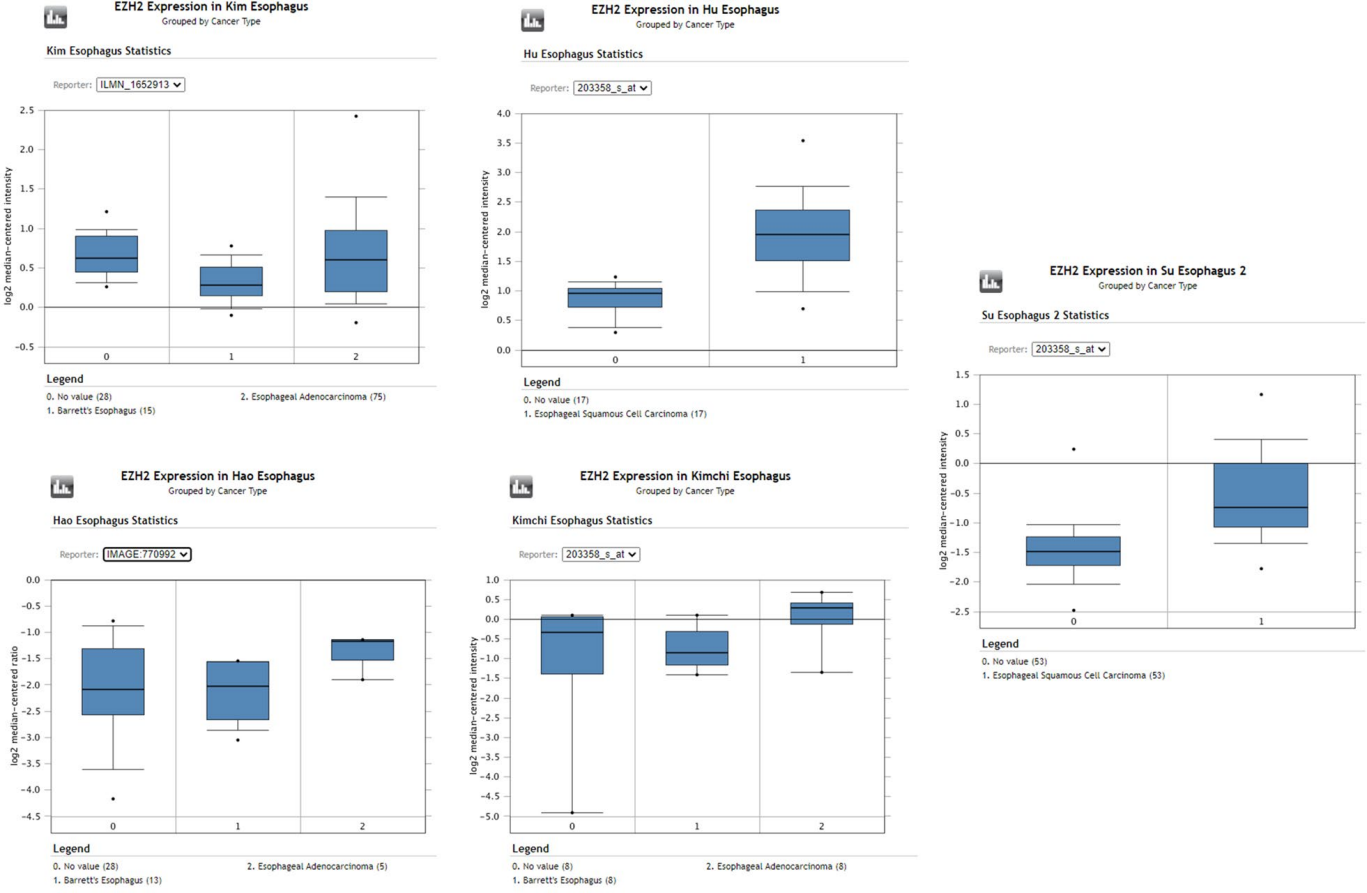
EZH2 expression from oncomine dataset. Box plots from Oncomine representing the higher EZH2 expression in Esophageal adenocarcinoma and Esophageal squamous cell carcinoma, compared to normal esophagus

Consistent with our findings various studies also reported overexpression of RUNX3 at mRNA level, thus pointing to a probable underlying mechanism of RUNX3 in the tumorigenesis of the esophagus. Hu dataset revealed an upregulation of RUNX3 in ESCC with a fold change of 2.661 (n = 34) Fig. 5 [38]. Another dataset of Su esophagus study and Kim esophagus study on 106 samples and 103 samples found RUNX3 overexpressing with a fold change of 1.48 and 1.29 respectively Fig. 5 [39, 40]. Some small patient dataset studies like Kimchi (n = 16) and Hao (n = 33) dataset also pointed out the upregulation of RUNX3 with a fold change of 2.337 and 7.741 respectively Fig. 5 [41, 42]. The expression of EZH2 was coincidentally also found to be overexpressing in tumor tissue in the same datasets considered earlier. Hu esophagus statistics showed EZH2 upregulated in tumor tissue by 2.09-fold Fig. 6 [38]. In Su esophagus study the fold change was 1.87 however, in Kim esophagus no change was seen in the expression Fig. 6 [39, 40]. Kimchi esophageal study and Hao esophagus study revealed EZH2 showing fold changes of 2.4 and 1.6 respectively Fig. 6 [41, 42].

## Discussion

RUNX3 is known to have tumor suppressive role in gastrointestinal cancers [17, 43]. Studies have shown low level of RUNX3 expression in esophageal tumor samples and its expression has been associated with radio-resistance and poor prognosis [33, 44]. Here, we investigated the status of RUNX3 in esophageal tumors from North Indian patients. Data revealed significantly upregulated mRNA of RUNX3 as compared to the normal adjacent tissue from the same patient in ~ 55% of the samples studied. This observation was statistically significant in the distribution of the expression values of the normal and the tumor tissue in this paired study. Notably, our data suggests that RUNX3 may not be always down-regulated in esophageal cancer, as demonstrated by several studies in different cancers [20, 26]. The observation of RUNX3 up-regulation in present study highlights its plausible role in esophageal cancer. Consistent with our study, oncomine data analysis also revealed RUNX3 and EZH2 up-regulation in five studies on esophageal cancer, Oncomine™ (Compendia Bioscience, Ann Arbor, MI) was used for analysis and visualization (Figs. 5, 6).

Correlating to the observed up-regulation of mRNA expression in tumors, RUNX3 protein level was also found to be upregulated in IHC of tumor compared to normal tissues. IHC data on tumor and control tissues thus further corroborated Real Time PCR. Since several reports suggested low RUNX3 expression in GI cancers, our data, adds a new dimension to the biology of RUNX3 and suggests that RUNX3 to function in tumor suppressive manner and emphasizes the need to revisit our understanding of RUNX3 biology in GI cancers. These results again points to the debate whether RUNX3 functions as a tumor suppressor gene or as an oncogene or can act as both depending on tumor context [45, 46]. Recently it has been demonstrated that RUNX3 when associates with MYC functions as a tumor promoter whereas; acts as a tumor suppressor when interacts with p53 [47]. Various other studies have demonstrated the oncogenic role of RUNX3 [46, 48–54].

The methylation experiments, consistent with the previous studies, demonstrated that RUNX3 expression correlated with the methylation status of the RUNX3 promoter CpG islands. In 27 cases, we found absence of methylation at the RUNX3 promoter and out of these 27 cases, 22 cases showed up-regulation of the RUNX3; *p* < 0.001 (Fig. 3; Table 3). Therefore, as suggested by previous studies, RUNX3 expression can be modulated by the differential methylation status at the promoter region [55].

To explain the presence of up-regulated RUNX3 in esophageal cancer of Indian patients, we conjecture two possible explanations. Mutations in RUNX3 may render inactivated or truncated version of the RUNX3 protein. The other explanation for RUNX3 up-regulation emanates from the possibility of adaptation of cells to over-express RUNX3, as a tumor suppressor gene, to counter the induction of cancer. It would be interesting to assess the structure and activity of RUNX3 protein (as transcription factor) in tumors where it is up-regulated to establish if its active or inactive in such cases. These, along with additional functional biology studies, may provide insights into the biological relevance of RUNX3 in esophageal cancers. Whittle et al. showed that in pancreatic cancer RUNX3 upregulation was involved in the increased metastasis, hence their study showed that RUNX3 played a role of tumor suppressor as well as tumor promoter in pancreatic ductal adenocarcinoma [46]. Similar studies in esophageal cancer are needed to establish a clearer role of RUNX3 on different characteristics of cancer cells. Another possible explanation can be attributed to dietary and environmental factors of our studied population leading to disparity in RUNX3 expression.

EZH2 is frequently over-expressed in a variety of cancers and its over-expression has been implicated in the down-regulation of RUNX3 [31]. However, the results presented here suggests that EZH2 doesn’t play a role in RUNX3 down-regulation and it’s the promoter methylation that regulates the expression of RUNX3. Interestingly, our results showed up-regulation of RUNX3 coincided with the absence of methylation of RUNX3 promoter region, suggesting that methylation of CpG islands of RUNX3 promoter regulate its expression, which is in agreement with other studies [56]. The observed positive correlation between RUNX3 and EZH2 (*p* < 0.03) suggests the possibility of their cooperative and/or interactive role in esophageal cancer, which invites further investigation. As cancer is a complex disease with multiple genes involved, it is always pertinent to consider that possibility of cooperative and/or interactive behavior of genes and their products in the pathogenesis of cancer, for identification of viable therapeutic targets.

## Conclusion

The results presented here highlights for the first time the relevance of RUNX3 and EZH2 in esophageal cancer, at least in Indian population. However, their aberrant expression in esophageal tissue biopsies also invite further investigation to be done to establish the role of RUNX3 in cancer is tumor suppressive or oncogenic.

## Data Availability

The datasets supporting the conclusions of this article are included within this article. Raw data are available from the corresponding author on reasonable request.
